# Children Are Back to School, but Is Play Still in Lockdown? Play Experiences, Social Interactions, and Children’s Quality of Life in Primary Education in the COVID-19 Pandemic in 2020

**DOI:** 10.3390/ijerph182312454

**Published:** 2021-11-26

**Authors:** Ana Lourenço, Fernando Martins, Beatriz Pereira, Rui Mendes

**Affiliations:** 1 Research Centre on Child Studies, Institute of Education (CIEC-IE), University of Minho, 4710-057 Braga, Portugal; beatriz@ie.uminho.pt; 2 Play Activity Department, Child Support Institute (IAC), 1050-185 Lisbon, Portugal; 3 UNICID-Applied Sport Sciences Research Unit (ASSERT), Polytechnic Institute of Coimbra, 3030-329 Coimbra, Portugal; fmlmartins@esec.pt (F.M.); rmendes@esec.pt (R.M.); 4 Polytechnic Institute of Coimbra, IIA, ROBOCORP, 3030-329 Coimbra, Portugal; 5 Instituto de Telecomunicações, Delegação da Covilhã, 6201-001 Covilhã, Portugal; 6 CIDAF, FCDEF, University of Coimbra, 3040-248 Coimbra, Portugal

**Keywords:** children, COVID-19, HRQOL, play, primary education, school, peers

## Abstract

The right to play is crucial for the overall development of children. Several studies highlight the need to have time and space to play, especially at school where children spend much of their time. Unfortunately, in formal education the obsession with academic achievements sidelines and ignores the importance of play. The neglection of play had already reached a critical stage before the pandemic, so data are needed to realize how the right to play in school is presently affected. This paper aims to understand children’s play experience in primary education during the pandemic. It investigates what activities children participated in and what materials were used, and provides insight into the social interactions between peers. Furthermore, children’s quality of life is explored. A group of 370 Portuguese children answered a questionnaire on play and social interactions, alongside with Peds 4.0^TM^ on health-related quality of life (HRQOL). The results showed that recess still emerges as a significant element of children’s daily lives, but COVID-19 has brought limitations on play experiences and peer-interaction. It might also have impacted HRQOL, especially in emotional functioning. Since play, health and well-being are closely connected, play opportunities at school are crucial in helping children to thrive in the pandemic, and should be invested in.

## 1. Introduction

Children’s right to play is stated in the Convention on the Rights of the Child (CRC) in Article 31: “1. States Parties recognize the right of the child to rest and leisure, to engage in play and recreational activities appropriate to the age of the child and to participate freely in cultural life and the arts.” [1] (p. 9). Furthermore, in 2013 a General Comment on the Right to Play was compiled, and several links between play and school context were stated, highlighting the importance of educational environments as play promotion settings [2]. Play in schools has been decreasing due to academic pressure; this restriction in play opportunities can constrain children’s opportunities for creativity, exploration, and social development [2].

Alongside the importance of the right to play in schools, we must highlight the significance of play to the overall development of children. Play develops children’s social and emotional skills and their ability to manage stress, and it promotes resilience and flexibility when facing uncertainty [3]. Evidence has shown that play, health, and well-being are closely connected [2,4,5].

When considering play opportunities in schools, recess comes to mind. Recess is one setting that can improve children’s experience, with studies dispelling the ancient ideas that the impact of recess on children’s lives was insignificant [6]. Studies have confirmed that recess also influences children’s academic achievements [7,8], as children’s behaviour, focus, and mood in the classroom improve as a result of recess [9]. Greater levels of attention and productivity are also reported as a result of recess [10]. Another crucial point of play at school is children’s peer interactions: recess can promote healthy relationships amongst children, as well as prevent bullying and social exclusion [7]. Children would rather play with friends than alone [11], and in this setting several competencies such as negotiation, cooperation, problem-solving, perseverance and self-control are developed [10].

Unfortunately, evidence has also shown that the potential of play during recess in schools is often ignored, with schools seldom investing in this. Even before the pandemic, school recess had already been facing challenges; professionals acknowledged the importance of recess, but shared the perception that children nowadays do not know how to play, and that the lack of activities, equipment, materials, and human resources leads, for instance, to moments of social conflict [7].

Fun is a key component when children evaluate their school recess; if children feel limited in their play opportunities, recess is perceived as boring, and therefore the renewal of play opportunities and materials is crucial [11]. Children consider being with friends and playing as their favorite features of recess and consider fighting and bullying as their least favorite [7]. Physically active games seem to be children’s favorite, but more sedentary options are also stated by some children, especially in scenarios where a lack of play opportunities or ideas is prevalent [11]. Both genders express a deep interest in active play. Girls demonstrate a wider range of activities at school recess, whereas boys prefer sports and activities that include intense physical exercise [6,12,13]. Children are also sensitive to the physical layout of the recess space, stating that wide-open spaces are better and that a space free of structures and without overcrowding is better [11]. Regarding spaces, previous research has shown that the sports field is the most used space by children during recess, followed by natural spaces. The most used play materials are balls, (especially by boys) and items brought from home, since schools seldom provide play materials [13].

In 2020, profound changes took place in children’s physical and social environments due to COVID-19, with the pandemic impacting different matters in children’s daily lives [14]. Children were deprived of going to school, one of the most important settings for their overall development. The first case of COVID-19 in Portugal appeared in March 2020, with the government closing primary schools from 16 March until mid-September 2020, opening for a new school year, and closing schools once again in the first months of 2021, totalling 24 weeks of closure [15].

Before the pandemic, children could move around during recess freely and could play with children from all classes. A lack of play materials was overcome by children bringing toys and games from home [13]. In the pandemic, following guidance from the General Directorate of Health and the General Directorate of Education, with some input from families and children, the changes made were as follows: children were organized into “bubbles”; interaction with other groups was avoided, namely, through different schedules for entrance, recess, and lunch; breaks between classes were sometimes reduced; spaces for each group, where children should stay at all times, were defined; circulation paths were established; play material brought from home was forbidden, and the use of items (for instance, play materials), should be carefully managed and for individual use only [16]. Educational institutions were deeply focused on these precautionary measures to combat the spread of the virus, overlooking the need to promote a rich physical environment that would support children’s activities and balance safety, risk-taking, and active play [17]. Children from other locations (e.g., Canada and other European countries) faced similar restrictions, with evidence showing that free movement was constrained at schools, with children confined to particular spaces for the entire week. This also lead to a change in the overall social patterns of children with their peers [18,19]. Experts have provided guidance around safe recess practices, and have also shared ideas to continue providing opportunities to play, e.g., creating bins of play materials to be used by each specific class, assuming a risk-benefit approach regarding active play [8].

The pandemic has also made an impact within research, with studies now focusing on collecting data on several matters that might have been impacted by the COVID-19 pandemic. For example, sleep and general behavior at home for small children [20], parental experience regarding pandemic restrictions and its impact on their children’s movement [21], and children’s independent mobility and physical activity [18], among others. Regarding play experiences in quarantine and restrictive environments, there is ongoing work suggesting that the right to play is impacted by restrictive environments, due to changes in access to play opportunities [22]. To our knowledge, research on education and COVID-19 mainly focuses on online learning processes, home-schooling, and inequalities, so data are needed on other elements; namely, children’s right to play in school. National (e.g., Portuguese General Directorate of Health and Portuguese Paediatric Society) and international institutions (e.g., UNESCO, International Play Association, and Right to Play) have requested that the right to play be considered, even in the pandemic [23,24,25]. The role of play in children’s coping and resilience mechanisms in uncertain and traumatic times is undeniable [26], and school’s recess as a place for children’s healing should not be overlooked [8]. This is particularly important since feelings of anxiety and depression among children are on the rise [27]. Actions to support children’s mental health, children’s play, and health promotion strategies are needed [18,27].

For this matter, data on health-related quality of life (HRQOL) could help professionals to adequately identify the activities and materials available at recess that promote HRQOL. Evidence confirms that children’s experiences in recess could be an indicator of HRQOL among school-aged children; more vigorous physical activities and the use of sports equipment relate to physical HRQOL, specific play-based movement relates to physical and emotional HRQOL, and playing on hard-surfaced areas relates to all HRQOL dimensions [6].

This paper aims to identify children’s play experiences in primary education during the pandemic, seeking to determine what activities children participated in, what materials were used, and what social interactions between peers are like today. Furthermore, links between the children’s quality of life and play activities at school are explored.

## 2. Materials and Methods

### 2.1. Study Design

The present study is an exploratory survey [28,29].

It was carried out in Lisbon Municipality, where the population survey carried out in 2011 showed that children’s population (0–14 years) increased by 9.4%. In 2020, the population of children in Lisbon represented 16.8% of the city’s inhabitants [30]. The data were collected in primary schools, from the parishes identified as having a high-density rate of children. The research was approved by the Ethical Committee of the University of Minho (CEICSH 080/2019). Informed consent was obtained from all participants by a form that was delivered by teachers to children’s legal guardians, usually parents.

### 2.2. Participants

The study involved 370 Portuguese children; 194 girls (52.4%) and 176 boys (47.6%), with a medium age of 8.65 years old (±0.794). Of these children, 190 were in the 3rd grade (51.4%) and 180 were in the 4th grade (48.6%). Their average number of siblings was 1.86 (±1.452).

Schools were selected by convenience (belonging to the same district and previously visited, to see if the attendance conditions were similar). The participants had to be, as inclusion criteria, attending primary school and in the 3rd or 4th grade. In Portugal, primary education is from 5–6 years, until around 10 years of age. Children attend school for a full day and usually have three periods of recess: two shorter periods in the morning and in the afternoon, and a longer period during lunch break.

### 2.3. Instruments

To address the children’s interactions and the use of school recess, we used a questionnaire entitled: “Children’s activities and interactions in recess” [31]. The questionnaire combined closed and open questions in 5 sections: (1) subject characterization; (2) opinions about recess; (3) activities and materials used; (4) adults’ presence during recess, and (5) peer interactions. There was no mention of the COVID-19 pandemic in the questions.

To collect data on children’s health-related quality of life (HRQOL), we used the Portuguese version 4.0 of the Pediatric Quality of Life Inventory (PedsQL 4.0^TM^) for children aged 8–12 years old [32,33]. The questionnaire had a total of 23 items answered against a 5-point Likert-type scale. The options were as follows: 0 = never a problem; 1 = almost never a problem; 2 = sometimes a problem; 3 = often a problem; 4 = almost always a problem. The scores were calculated following the instructions given by the author, ending up with a global health quality score, a psychosocial health score, a physical health score, and scores on 4 separate dimensions (physical, emotional, social, and school). The closest the score was to 100, the better the score. In the present study, the questionnaire’s internal consistency was adequate (Cronbach’s alpha = 0.827).

Both questionnaires were self-answered by children in late-October/early-November 2020, in the school context.

### 2.4. Statistical Analysis

Children’s answers regarding their play experience, social interactions, and health-related quality of life in school during the pandemic were analyzed by descriptive statistics [34,35], with IBM SPSS Statistics (version 26, USA).

For the open-ended questions, data were coded [36] and categorized in a posteriori theme, with the support of NVivo software (QSR, Melbourne, Australia). In the results section, the themes are presented for each question, alongside the frequency of each one. The findings are illustrated with children’s quotes, and identified by gender and age.

## 3. Results

### 3.1. Children’s Play Experiences

Children’s play experiences in school during the pandemic can be seen in Table 1.

Most children stated that they enjoyed recess (89.4%). We identified several categories for this opinion; namely, children felt they could “play”, “have enough time” for their activities, “have fun”, “talk and meet with friends”, do “specific activities”, take a moment to “relax”, enjoy “feeling free outdoors”, and “be with adults” (Appendix A, Table A1).

- The primary reason as to why recess was enjoyed, given by more than half of the children (56.51%), was that it is the time to play: “classroom is to work, recess is to play” (girl, 9 years old).
- The next reason (12.72%) was that recess provides enough time for children’s activities: “it is suitable to play and eat something” (girl, 9 years old).
- The third reason (12.53%) was that it is fun: “I have lots of fun with my friends” (boy, 8 years old).
- Other reasons mentioned were as follows: 5.92% included talking and meeting with friends: “it’s the time when I can talk with my friends” (girl, 8 years old); 4.44% said they like to do specific activities “we can play hide-and-seek and go catch and other things” (girl, 9 years old); 3.85% mentioned recess as an opportunity to relax and take a break: “we can take a break from work” (girl, 9 years old); 3.55% identified feeling free and said they enjoyed being outdoors: “not being in the classroom all day” (girl, 8 years old); finally, 0.59% identified interaction with adults as what makes them like recess: “the staff take good care of us” (boy, 9 years old).

When considering the reasons why almost 11% of children stated that they did not enjoy recess, three main issues surfaced: “not enough time”, “recess conditions”, and “COVID-19” (Appendix A, Table A2).

- The majority of children (78.05%) mentioned that they do not have enough time for recess, in terms of both the small breaks during the day (“the first break in the morning should be longer” (boy, 7 years old)) and the lunch break (“we play for an hour but I would like it to have more time” (girl, 10 years old)). The feeling was that time goes by too quickly: “when we are at recess, it feels like only 5 min have passed by and we are already back to the classroom” (boy, 9 years old).
- Other children (12.20%) described certain recess characteristics as the reasons they did not like it, such as the size of the playground (“I would like to make the recess area bigger”, (boy, 9 years old)); weather conditions (“sometimes it is cold outside and I would rather stay in the classroom” (girl, 9 years-old)); structural barriers “because of the poles and the concrete floor” (girl, 9 years old), or simply because they felt that they “have nothing to do” (girl, 9 years old).
- With regard to the constraints due to COVID-19, 7.32% of the children mentioned a reduction in the time allocated to recess (“before we had 2 h, now it is only one” (boy, 8 years old)), in addition to being confined to a specific location in recess (“we are only allowed to be in the football field” (girl, 10 years old)). One child even stated, “with the virus we cannot play” (girl, 9 years old).

It is not surprising to realize that children, despite enjoying recess, still wish to have more time for it (75.4%). On the other hand, about a quarter of children (24.6%) stated that they do not want more time for recess, because they like and want to learn in the classroom.

When asked if there were activities that children would like to do at recess but are not allowed to, about half of the children stated “yes” (46.6%), and slightly more than half stated “no” (53.4%).

In the positive answers (Appendix A, Table A3), we identified the following themes in children’s answers: “sports and movement activities”; “playground equipment”; “toys and games”; “traditional play”; “arts and music activities”; “technologies”; “social interactions”, and “natural elements play”.

- The activities children most commonly stated that they were unable to do at recess were sports and movement activities (33.68%): “gymnastics with my friends” (girl, 9 years old); “[playing] soccer more” (boy, 8 years old); “play fighting” (boy, 9 years old), and “walking around in the whole school” (boy, 9 years old).
- The following activities connected to playground equipment were mentioned (15.79%): “[going] up the slide” (girl, 8 years old) and “[riding] the swing” (girl, 8 years old).
- The third category refers to restrictions relating to toys and games (13.16%): they “cannot bring toys to school” (boy, 8 years old), cannot play with “draughts [or] puzzles” (boy, 9 years old), and cannot “play with dolls” (girl, 8 years old).
- Some children mentioned traditional play as being restricted (12.11%); one stated, “I would like to [jump rope]” (girl, 9 years old), and one said there is “no hopscotch painted on the floor” (girl, 9 years old).
- Arts and music activities were in some cases not allowed (10.53%), including “drawing” (girl, 9 years old), “[listening] to music” (boy, 8 years old), and “different dances” (girl, 9 years old).
- Technologies were mentioned by 6.32% as being forbidden, including “[bringing] mobile to school” (boy, 10 years old) and playing “electronic games” (girl, 9 years old).
- Finally, some children mentioned social interactions (3.16%) and natural play (2.11%) as forbidden, including “[playing] with other classes” (boy, 10 years old), “[touching] other people” (girl, 9 years old), and “climbing trees” (girl, 9 years-old).

Regarding the reasons for the restrictions (Appendix A, Table A4), a “lack of materials or space”, “school rules and guidelines”, and “COVID-19” were the categories that arose from children’s answers.

- Regarding children who mentioned a lack of materials (39.13%), children identified either materials that cannot be taken outside, or the non-existence of materials. They stated that: “we cannot bring paper sheets outside” (girl, 9 years old), and “school does not have balls and if I bring mine they take it” (boy, 8 years old). Regarding spaces (namely, playground equipment), one child mentioned, “that equipment does not exist in my school” (girl, 9 years old).
- Some statements were made regarding the school’s rules and guidelines (33.04%): “we cannot write on the floor at the school” (girl, 8 years old), “only children from the kindergarten can play there” (girl, 10 years old), and “adults say it is forbidden” (girl, 9 years old).
- Lastly, in more than a quarter of all answers (26.96%), COVID-19 restrictions were mentioned: “in the pandemic, we cannot bring toys to school” (boy, 9 years old), “the coronavirus is in the air” (girl, 9 years old), and “we cannot touch each other” (girl, 8 years old). Only children’s answers that clearly mentioned COVID-19 were inserted here, but it might be an underestimated value, as some answers coded in the previous category (school rules and guidelines) could also be connected to the pandemic.

Regarding the materials used in recess (Appendix A, Table A5), balls were mentioned most frequently (18.6%), followed by skipping ropes (13.9%) and hula hoops (12.8%). Some children did not use any materials for their activities (11.7%).

With regard to the activities carried out in recess (Appendix A, Table A6), running was the most preferred (15.15%), followed by hide-and-seek (13.03%) and talking (12.51%). “Other” answers included human football, reading, “Simon Says”, and play activities created by the children.

On the subject of favorite spaces (Table 2), the playground was the most popular (29.9%), followed by “more than one” play space (20.4%) and “other” locations (18.8%). Regarding the most-used space, the playground and the games and sports fields were most common (26.6% each), followed by “more than one” location (25.4%). “Other” answers included covered spaces, roads, and ramp areas.

As first and second favorite activities (Table 3), children chose playing football (27.2%) and “other” activities (28%), followed by hide-and-seek (19.3%). In “other”, the children mentioned mainly physical activity games, such as dancing and drawing.

### 3.2. Children’s Social Interactions

Children’s social interactions in primary school during the pandemic, in relation to playing alone, can be seen in Table 4.

Almost all children shared that they did not play alone at recess (92.3%), and most of them also stated that they were never left alone during recess, due to other children not wanting to play with them (70.3%). We must highlight that 36.5% of the children who answered “no” gave inputs in the questionnaire’s following question, which asked how they felt when that situation happened; therefore, we are uncertain if the number of negative answers truly shows the reality experienced by children.

Considering the reasons why most children did not play alone (Appendix A, Table A7), only two themes arose from the answers: “having friends” and “not liking it”.

- Most children identified friends as the main reason for not playing alone (88.50%): “friends ask me to play with them” (girl, 9 years old); “my friends never leave me by myself” (girl, 8 years old). The number of friends was either high or restricted to close friendships, but in both cases, children felt accompanied while playing: “I have lots of friends” (boy, 8 years old); “I have a best girl-friend and a best boy-friend” (girl, 9 years old). A child even stated, “a life without friends is not worth living” (boy, 9 years old).
- Feelings of sadness and boredom were the main reasons for children not liking to play alone (11.50%): “I feel sad if I play alone” (boy, 9 years old); “[playing] alone is boring” (boy, 9 years old).

The wish of having more friends (Table 5) was balanced between the answers “yes” and “no” (50.4% and 49.6%, respectively).

We looked more closely at the reasons why half of the children would like to have more friends (Appendix A, Table A8). The themes that arose were “[meeting] new people”, “[playing] more”, “[having] more fun”, having “few friends”, experiencing “feelings of loneliness”, and “restrictions due to the pandemic”.

- Most children would like to have more friends, since they like to meet new people (42.59%); some stated that “it is always nice to have new friends” (girl, 9 years old) and that they like “to meet new people and get more friendships” (girl, 9 years old). Children also stated that having more friends would help in certain cases: “when my friends don’t come to school I can play with others” (boy, 7 years old); “if some children don’t want to play with me I could play with the others” (boy, 8 years old).
- For some children (20.37%), making new friends would allow them to play more, either by engaging in different activities or by expanding the play experience: e.g., “to [play] new games” (boy, 9 years old), “for games to be bigger” (girl, 8 years old), and “to make more games” (boy, 11 years old).
- Children identified having more friends as a way to have more fun (19.44%): “it would be more fun” (boy, 8 years old); “playing with only one friend sometimes isn’t that much fun” (girl, 9 years old).
- Having few friends (7.41%) and feelings of loneliness (5.56%) were other reasons for wanting more friends: “I only play with two friends” (boy, 7 years old); “I feel a bit lonely” (girl, 9 years old).
- Restrictions in peer interactions due to COVID-19 (4.63%) were the last reason stated by children: some stated they wished “to be able to be with friends from the 3rd grade” (girl, 8 years old); “we cannot be with children from other classes” (girl, 9 years old). A child even stated, “I think children should be allowed to play with other children” (girl, 9 years old).

With regard to the number of friends the children had (Table 6), most children had 3–4 friends in their class (40.8%). Regarding friendships with children from other classes, most mentioned 1–2 friends (40.4%), and a high percentage stated that they did not have friends in other classes (35.6%). From that number, some gave COVID-19 restrictions as the reason for this (10.3%).

### 3.3. Children’s Quality of Life

The health-related quality of life (HRQOL) and the scores of the different dimensions of functioning can be seen in Table 7. The global score was 77.77; regarding the four dimensions, physical and social functioning had higher scores (81.65 and 81.24), and emotional and school functioning had lower scores (72.49 and 72.83).

## 4. Discussion

The present study sheds light on how children’s play activities in primary education settings have been experienced during the pandemic, by providing children’s own perspectives, a point identified in the literature [22].

From our results, recess still emerges as a very important element of children’s daily lives, with most children enjoying recess because they have opportunities to play, have fun, and be with their friends, which is confirmed by previous research [7,11]. In previous research, bullying and peer conflict [7] were the main reasons stated for not enjoying recess. Our study confirmed that the children felt that recess was not long enough and, since COVID-19 restrictions have appeared, that the time to play had been shortened, and that there were limitations on free movement.

The lack of materials, as well as school guidelines and rules, were cited as reasons why more than half of the children were not doing certain activities that they would like to do. Moreover, many children justified restrictions with “it is forbidden”, which leads us to believe that children may not understand the reasons behind the rules or guidance. This is particularly important to prevent disruptive behaviors and feelings of injustice. However, the pandemic has left its mark in this matter. About a quarter of the children shared that the constraints in their play experience were connected to COVID-19; namely, with regard to bringing toys and games to school, moving around during recess, and interacting with their peers. The lack of play material available in schools was already a reality that existed before the pandemic [7], but it seems clear that this was aggravated by COVID-19 restrictions. Prior studies have identified that items brought from home are one of the most used materials by children during recess [13], and therefore schools should be aware that investment in play materials is critical. Schools should not overlook the need to have a rich play environment [17] and should continue providing play opportunities for children; namely, by prioritizing the suggestions of creating bins of equipment [8] for each class.

Considering the activities that are not allowed, sports and other physical play were the first activities that seem to have been restricted. This type of play should be encouraged, considering the need to promote healthy lifestyles during childhood to prevent child obesity and a sedentary lifestyle [37], and promote HRQOL [38]. The evidence before the pandemic is clear in stating that children did not achieve the recommended daily amount of physical activity [37]. Research on children’s physical activity in the pandemic showed that the lack of physical activity worsened after the pandemic, with children showing a decrease, as a result of confinement and social isolation [39,40]. Teachers and other school professionals should be supported to allow active play opportunities to happen for children, and to ensure administrative and financial support from the school’s management [41].

With regard to social interactions, the findings are consistent with previous research, with most children preferring to play with friends rather than be alone [11]. Nevertheless, about half of the children would like to have more friends, as they enjoy meeting new people and feel it would allow them to play more and to have more fun. The establishment of new relationships between peers could be compromised, since social interactions have suffered deep changes with the pandemic [19]. This is particularly relevant since healthy relationships, promoted by peer interaction at recess, prevent bullying and exclusion [7] and stimulate a series of interpersonal competencies [10]. Furthermore, the children who had already mentioned having few friends and feelings of loneliness could experience reduced opportunities to socially interact.

We should be particularly attentive to the messages we provide children regarding the pandemic. Some children seem to be afraid of touching people or materials, which, alongside the changes in children’s daily lives, can contribute to an increase in mental health issues, such as anxiety and depression, in childhood [42]. We found that, regarding HRQOL, the dimension that had the lowest score was emotional functioning. Experts suggest that children, when returning to school, might show some behavioral changes (e.g., difficulties in self-regulation and conflict management as well as aggressive or withdrawal behaviors), and school professionals should play a key role in supporting children to overcome these challenges [8]. This should be further investigated, to combat the collateral damage caused to mental health as a result of the COVID-19 pandemic. Children still enjoy recess, which shows their ability to make the most of every opportunity, as well as their natural inclination to play [43]. Alongside the lack of material and school guidelines, COVID-19 is now perceived as another reason why children see their play experience at school constrained. Before the pandemic, play as a child’s right was already frequently overlooked [2]; it seems that our results imply that the right to play at school is under threat. The pandemic could be used as an excuse not to invest in recess, thus limiting children’s experience and opportunities, and this could cause severe damage in children’s overall development.

The information we currently have regarding the guidelines for the next school year (2021–2022) allows us to state that the focus is still on COVID-19 management, with little focus on children’s right to play and its recovery. The General Directorate of Health and the General Directorate of Education have kept the guidelines that were in place last year (e.g., preventing contact between classes) and reviewed only the rules for quarantine and management of positive cases in schools [44]. On the other hand, the Portuguese Ministry of Education released a plan for recovery that highlights the need for social and emotional support for children, both during the pandemic and post-pandemic [45].

Despite the limited available evidence linking the lack of play as a result of quarantine and the pandemic restrictive environments to children’s overall health [22], it is an unquestionable fact that children express their emotions and fears through play, especially in environments that are supportive [43]. In future research, data collected on children’s play experiences should include children’s views of improvements to recess enjoyment during the pandemic. Children’s opinions about the changes that were made in recess due to the pandemic are key. Research is needed on these opinions regarding activities and equipment management, and the involvement of children in recess planning and organization should be promoted [8]. Deep knowledge of the school’s play environment, as perceived by children, is crucial in the design of future intervention programs aimed at restoring children’s well-being [14] and recess’ potential to promote overall health and well-being. Furthermore, there are two points that our study does not address, which should be taken into account in future studies: the gathering of data from other stakeholders, such as teachers, school staff and school directors; and the use of multiple sources to achieve an in-depth knowledge of the children’s reality (e.g., combine qualitative and quantitative sources of information).

## 5. Conclusions

Educational environments are crucial in promoting the right to play. Precautionary measures imposed to curb the spread of the COVID-19 virus have limited play experiences, play material management, and social interactions. Children continue to experience a semi-lockdown situation regarding play opportunities at school. This is particularly disturbing since play, health, and well-being are closely connected. Children’s overall development will be detrimentally affected as a result of confinements, quarantines and social distancing measures. Furthermore, thinking and learning are heavily affected by stress. However, the well-being of children can be restored if children can play, socially interact with peers, and rest [8]. Schools must be aware of the importance of continuing to provide play opportunities for children, aiming not only to increase school engagement, to improve mental health, and to increase learning [8], but also to protect and enforce children’s rights [1].

## Figures and Tables

**Table 1:** Children’s play experiences in primary school during the pandemic.

Items	Yes		No	
	*n*	%	*n*	%
Do you like recess time?	328	89.4%	39	10.6%
Would you like to have more time?	270	75.4%	88	24.6%
Is there something you cannot do?	159	46.6%	182	53.4%

**Table 2:** Favorite and most-used spaces in primary school recess in the pandemic.

Favorite Space			Most-Used Space		
	*n*	%		*n*	%
Playground	97	29.9%	Playground	89	26.6%
More than one space	66	20.4%	Games and sports field	89	26.6%
Other	61	18.8%	More than one space	85	25.4%
Games and sports field	57	17.6%	Other	43	12.8%
Nature	23	7.1%	Nature	12	3.6%
Painted floor games	11	3.4%	Painted floor games	13	3.9%
Don’t know	9	2.8%	Don’t know	4	1.2%
Total	324	100%	Total	335	100%

**Table 3:** First and second preferred activities in primary school recess in the pandemic.

First Favorite Activity			Second Favorite Activity		
	*n*	%		*n*	%
Playground	19	5.6%	Playground	16	5.1%
Floor games	6	1.8%	Floor games	11	3.5%
Play catch	38	11.1%	Play catch	27	8.7%
Football	93	27.2%	Football	31	10.0%
Running	42	12.3%	Running	27	8.7%
Inventive play	37	10.8%	Inventive play	33	10.6%
Hide-and-seek	30	8.8%	Hide-and-seek	60	19.3%
Rope skipping	20	5.8%	Rope skipping	19	6.1%
Other	57	16.7%	Other	87	28.0%
Total	342	100%	Total	311	100%

**Table 4:** Children’s social interactions in primary school in the pandemic—playing alone.

Items	Yes		No	
	*n*	%	*n*	%
Do you usually play alone at recess?	27	7.7%	324	92.3%
Have you ever been alone at recess?	103	29.7%	244	70.3%

**Table 5:** Children’s social interactions in primary school in the pandemic—new friendships.

Items	Yes		No	
	*n*	%	*n*	%
Would you like more friends to play with?	173	50.4%	170	49.6%

**Table 6:** Number of friends in primary school in the pandemic.

Number of Friends		Class		Other Classes	
		*n*	%	*n*	%
No mention	Without reason	1	0.3%	74	25.3
	COVID-19 reason	0	0.0%	30	10.3
1–2 friends		71	21.8%	118	40.4
3–4 friends		133	40.8%	47	16.1
More than 5 friends		73	22.4%	13	4.5
Everybody		48	14.7%	10	3.4

**Table 7:** Children’s health-related quality of life in primary school during the pandemic.

Peds 4.0 ^TM^ Global and Dimension Scores			
	*n*	Mean	sd
HRQOL Total	338	77.77	12.70
Physical Health	340	81.65	15.36
Psychosocial Health	337	75.71	14.04
Physical Functioning	340	81.65	15.36
Emotional Functioning	340	72.49	19.90
Social Functioning	338	81.24	18.56
School Functioning	339	72.83	16.91

## Data Availability

No new data were created or analyzed in this study. Data sharing is not applicable to this article.

## Appendix A

**Table A1:** Reasons stated for liking recess.

Categories		
	*n*	%
To play	191	56.51%
Enough time for activities	43	12.72%
To have fun	42	12.43%
To talk and meet friends	20	5.92%
To do a specific activity	15	4.44%
To relax	13	3.85%
To be free	12	3.55%
To be with adults	2	0.59%

**Table A2:** Reasons stated for not liking recess.

Categories		
	*n*	%
Not enough time	32	78.05%
Recess conditions	5	12.20%
COVID-19	3	7.32%
Other	1	2.44%

**Table A3:** Restrictions in recess.

Categories		
	*n*	%
Sport and movement activities	64	33.68%
Playground equipment	30	15.79%
Toys and games	25	13.16%
Traditional play	23	12.11%
Arts and music activities	20	10.53%
Technologies	12	6.32%
Social interaction	6	3.16%
Natural elements play	4	2.11%
Others	6	3.16%

**Table A4:** Reasons for restrictions in recess.

Categories		
	*n*	%
Lack of material or space	45	39.13%
School rules and guidelines	38	33.04%
COVID-19	31	26.96%
Others	1	0.87%

**Table A5:** Materials used by children in primary schools’ recess in the pandemic.

Materials		
	*n*	%
Balls	138	18.6%
Ropes	103	13.9%
Hula hoops	95	12.8%
None	87	11.7%
Other	65	8.7%
Water	49	6.6%
Dirt	48	6.5%
Rocks	46	6.2%
Elastic	39	5.2%
Cards	37	5.0%
Toy cars	17	2.3%
Dolls	12	1.6%
Radio	6	0.8%
Electronic devices	1	0.1%
Total	743	100%

**Table A6:** Activities carried out by children in primary schools’ recess in the pandemic.

Activities		
	*n*	%
Running	287	15.15
Hide-and-seek	247	13.03
Talking	237	12.51
Football	173	9.13
Dance	118	6.23
Hopscotch	117	6.17
Play catch	113	5.96
Drawing	100	5.28
Rope skipping	99	5.22
Playfighting	71	3.75
Other	62	3.27
Music	58	3.06
Skipping elastic	44	2.32
Cards	37	1.95
Puzzles	35	1.85
Marbles/Caps	26	1.37
Symbolic play (doctors, houses)	22	1.16
Draughts	18	0.95
Whipping-top	14	0.74
Eletronic games	10	0.53
Loto	7	0.37
Total	1895	100%

**Table A7:** Reasons for not playing alone.

Categories		
	*n*	%
Having friends	177	88.50%
Not liking	23	11.50%

**Table A8:** Reasons for wanting more friends.

Categories		
	*n*	%
Meet new people	46	42.59%
Play more	22	20.37%
To have more fun	21	19.44%
Few friends	8	7.41%
Feelings of loneliness	6	5.56%
Restrictions from COVID-19	5	4.63%
